# Use of pregnancy ultrasound before the 19th week scan: an analytical study based on the Icelandic Childbirth and Health Cohort

**DOI:** 10.1186/s12884-018-2134-1

**Published:** 2018-12-29

**Authors:** Kristine Flo Halle, Maria Fjose, Hildur Kristjansdottir, Amalia Bjornsdottir, Linn Getz, Margret Olafia Tomasdottir, Johann Agust Sigurdsson

**Affiliations:** 1 General Practice Research Unit, Department of Public Health and Nursing, Norwegian University of Science and Technology (NTNU), Håkon Jarls gate 11, NO-7491 Trondheim, Norway; 2 Department of Midwifery, University of Iceland, Reykjavik, Iceland; 3 Department of Teacher Education, University of Iceland, Reykjavik, Iceland; 4 Department of Family Medicine, University of Iceland, Reykjavik, Iceland, and Centre of Development, Primary Health Care of the Capital Area, Reykjavik, Iceland

**Keywords:** Pregnancy, Prenatal screening, Combined test, Informed choice, Ultrasound, Medicalization

## Abstract

**Background and aim:**

Use of ultrasound scans early in pregnancy is increasing, but we have limited knowledge about the actual prevalence, associated decision-making and impact on expectant women/couples in a general population. The aim of this study was to document the use of, and experiences related to, foetal scanning *before* the recommended 19th week scan among pregnant women in Iceland.

**Population and methods:**

The data come from the Icelandic Childbirth and Health Cohort Study 2009–11. A total of 1111 women attending prenatal care at primary care health centres answered questionnaires before mid-pregnancy and after birth, including questions about the number of scanning procedures during pregnancy. These might include consumer-initiated ‘pregnancy confirmation scans,’ scans for clinical reasons, and screening for foetal anomalies in week 11–14 which is optional in Iceland. The questionnaires also addressed parental decision-making associated with the 11–14 week screening, perception of the pre-screening information, reasons for attending or declining, and whether/how early foetal screening affected the women’s concerns related to the unborn child.

**Results:**

A total of 95% of the women reported some kind of foetal ultrasound scanning before the 19th week scan, and 64% reported two or more scans in this period. 78% of the women chose to participate in screening for foetal anomalies in week 11–14. Decision-making in relation to this screening was mainly informed by sources outside the healthcare system, and many women characterized participation as ‘self-evident’. Most women felt they got sufficient information about the scope of screening, whilst information regarding potential downsides and risks was frequently perceived as insufficient. Most women who chose the 11–14 week screening reported a reassuring or neutral effect, whilst 10% of the women reported that it increased their concerns related to their unborn child.

**Conclusions:**

Ultrasound scans in the first half of pregnancy are in high use in Iceland and have apparently become part of a broader pregnancy culture, encompassing both high- and low-risk pregnancies. Whether this is a favourable development or to some extent represents unwarranted medicalization, can be debated. More balanced information might be provided prior to early screening for foetal anomalies.

## Background

Ultrasound scans play an increasing role in antenatal care and culture in industrialized societies. The purpose of such scanning can be complex. For many expecting parents, confirmation of the pregnancy and a first ‘encounter’ with the child-to-be are a central motivation. From the service provider’s point of view, the most immediate aim is to confirm a viable pregnancy. Beyond this, one focus is on pregnancy-related risk factors, in particular determination of the term, number of embryos and location of the placenta. Another focus might be to examine the foetus for potential malformations, diseases or syndromes, including Trisomy 21 (Down’s syndrome) [1]. Systematic foetal screening typically involves visual examination of the foetus by ultrasound and measurement of foetal nuchal translucency (NT), often combined with measuring certain biomarkers in maternal blood in pregnancy week 11–14 (“combined test”). A more recent method involves examination of free foetal DNA in maternal blood, so-called Non Invasive Prenatal Testing (NIPT) [2]. Due to the diversity and complexity of prenatal testing, provision of information prior to scanning/testing is a demanding issue [3, 4].

## Why do women attend ultrasound scans in the early phases of pregnancy?

Several studies have shown that visualization of a living, thriving foetus might represent the most prominent motivation for attending an ultrasound scan, in addition to confirming and dating the pregnancy [5–7]. Since the scan as such has strong appeal to many people, it can be hard to disentangle its biomedical purposes from the human wish to simply ‘see’ the foetus. Scanning technology might thereby contribute substantially to shaping the culture surrounding even low-risk pregnancies [8].

## Foetal screening and informed participation

Many countries have introduced national screening programmes for foetal anomalies at an affordable price or free of charge [9]. The well-established scan around pregnancy week 19 is in several countries, including Iceland, supplemented by an offer of *early* pregnancy screening in week 11–14 (most often the combined test) [10]. The test sensitivity for Down‘s syndrome is 90–95% [11, 12]. In the presence of increased risk, the pregnant woman is offered an invasive diagnostic procedure, which carries approximately 1% risk for unintended pregnancy loss. The cut-off point for offering invasive testing is typically 1/250 or higher [11].

Prenatal screening involves complex technology and is inherently value-laden [3–6, 13, 14]. As in all screening programmes, potential participants are entitled to correct, understandable and balanced information, so as to enable informed participation [15].

## Current practice in Iceland

At the time of the present study, Iceland had 320,000 inhabitants, with 70% living in the greater capital area. Then and now, the Icelandic healthcare system resembles the other Nordic Welfare states with some variations [16, 17]. Public primary healthcare is intended to represent the entry point in most instances. There is, however, no systematic referral or gatekeeping system [18], and private specialists can be directly sought for a relatively affordable fee.

Icelandic primary care, including maternity care, is carried out at public healthcare centres staffed by GPs, midwives, nurses and other auxiliary staff, sometimes including consulting obstetricians (publicly employed). Maternal care in accordance with Icelandic guidelines is free of charge. It is to a large extent delivered by the primary care midwives, starting no later than pregnancy week 12 [19]. In case of risk pregnancies, primary care staff collaborate closely with hospital obstetricians. The guidelines in Iceland have since 2008 recommended one ultrasound scan in week 19–20 (the 19th week scan). When pregnant women enter the primary healthcare system for their first visit, they should however, according to the same guidelines, be asked if they want information about early pregnancy screening for foetal anomalies (the combined test, week 11–14). Such screening is done at the National University Hospital in Reykjavik or Akureyri hospital in North Iceland. Pre-test information is available both in primary care and at the screening site [19]. As early foetal screening is not directly recommended in the Icelandic guidelines, it is not free of charge, but costs around 93 Euros [20].

As previously explained, Icelandic women who become pregnant have, since before this study started, easy access to private gynaecologists who employ ultrasound equipment as part of their clinical routines. A high number of women consequently have their pregnancies confirmed by a scan, before signing up for public antenatal care [21]. The cost for such a consumer-initiated specialist consultation with ultrasound scanning has a maximum fee of 117 Euro (in comparison, a primary care consultation with a GP costs 10 Euro, the situation in the study period was comparable). The content of the private consultations in early pregnancy may evidently vary. It can be assumed that a confirmatory scan is performed, and that information about the optional 11–14 week screening programme is provided. Beyond this, we have limited knowledge about the expectant parents` ideas about, and experiences with, ultrasound in the early phases of pregnancy, from a general population perspective. We also lack knowledge about the parents’ information level and decision-making related to the foetal screening programme in week 11–14.

The aim of the present study was to explore the overall prevalence of prenatal ultrasound scanning before the recommended foetal scan in week 19, including scans related to screening in week 11–14, among pregnant women in Iceland. Furthermore, we explored where the women sought/got information about prenatal screening, the parental decision-making process, perception of the information received, and the impact of screening on their concerns related to the foetus` health.

## Methods

Our data come from the Icelandic Childbirth and Health Study (C&H study) in 2009–2011. Its design and questionnaires were based on a similar cohort study on pregnant women carried out in Sweden 1999–2000, *Kvinnors upplevelse av barnafödande* (the“KUB”study) [22–24]. It is a population-based cross-sectional cohort study of pregnant women attending routine antenatal care at primary healthcare centres. We used consecutive convenience sampling methods, stratified according to residency. This was to attain a distribution similar to the entire country, with a ratio of 70:30 for urban versus rural residency. The study is described in more detail elsewhere [23].

Participating women answered a comprehensive questionnaire around pregnancy week 16 (phase I). A total of 1111 participants replied, constituting 63% of the 1765 women who initially agreed to receive the questionnaire (convenience sampling method). This constituted 23% of all childbirths in 2009. Phase II was conducted 5–6 months postpartum (765 participants responded), and phase III 18 to 24 months after delivery (657 participants, i.e. 59% of the original 1111 sample). The present analysis is based on phases I and II.

The survey questions included socio-demographic and obstetric background and use of health care services, including: ultrasound scans and screening for foetal anomalies, perception of received information, decision-making related to prenatal tests, emotional well-being and pregnancy-related concerns. Education was categorized as: comprehensive school covering 10 years (primary), upper secondary schools 3–4 years after comprehensive school (secondary), and technical education/university less than 4 years and university more than 4 years (higher education).

To test the general validity of the dataset, evaluation was done for possible self-selection bias caused by dropout after phase I, as recommended by Fewtrell et al. [25]. This analysis showed that those who participated in both phases I and II were at baseline older (*p* < .001) and with a somewhat higher educational level (*p* = .001), compared to those who participated only in phase I. No difference was found regarding residency, civil status or parity.

## Statistical analysis

We used Statistical Package for Social Sciences (SPSS) for Windows, version 24.0. Descriptive data are presented as frequency and percentage. We used Pearson’s Chi-Square test to assess significance between groups on demographic variables. Binary logistic regression analyses were used to estimate the association between groups, using crude odds ratio (OR) with 95% confidence intervals (CI). We considered a two-tailed value of *p* < .05 to be significant.

## Ethics approval and consent to participate

The study was approved by the National Bioethical Committee in Iceland (VSNb2008010023/03–1), and reported to the Data Protection Authority (S3695/2008 LSL/). The study was also approved by the professional authorities of the involved health care centres. Participant consent was not required for this analysis, as it is based on a previously de-identified database for which informed consent was obtained according to the Icelandic Bioethics Committee.

## Results

Socio-demographic characteristics of the participants at entry are shown in Table 1. Most participants were well-educated, with 63% having higher education (more than 14 years in total). A total of 69% lived in the capital area, and 93% lived with a partner (data not shown in table).

**Table 1:** Characteristics, obstetrical history and type of health care providers among participants at their first contact with the health care service. Percentages (absolute figures within brackets), odds ratio (OR) and 95% confidence intervals (95%CI) for those who chose combined prenatal screening test for foetal anomalies in the Childbirth and Health Study, Iceland

	All (Number)	%	Combined prenatal screening test at week 11–14			
			%	OR	95%CI	*p*-value
Total	1111		78.3 (862/1101)			
Parity at study entry						
Primipara	439	39.5 (439/1110)	82.3 (359/436)	1	Ref.	
Multipara	671	60.5 (671/1110)	75.6 (503/665)	0.67	0.49–0.90	.009
Education						
Primary (10 years)	123	11.1 (123/1109)	73.0 (89/122)	0.53	0.34–0.83	.006
Secondary (4 years**)**	291	26.2 (291/1109)	67.9 (195/287)	0.42	0.30–0.58	.009
Higher (total > 14 years)	695	62.7 (695/1109)	83.5 (577/691)	1	Ref.	
Age (years)						
18–24	186	16.7 (186/1111)	75.0 (138/184)	0.39	0.23–0.69	.001
25–34	733	66.0 (733/1111)	76.5 (557/728)	0.43	0.27–0.69	<.001
> 35	192	17.3 (192/1111)	88.4 (168/190)	1	Ref.	
Region						
Urban	763	68.7 (763/1110)	86.5 (656/758)	1	Ref.	
Rural	347	31.3 (347/1110)	60.1 (206/343)	0.23	0.17–0.32	<.001
I have tried to become pregnant for more than one year						
Yes	147	13.3 (147/1106)	83.0 (122/147)	1	Ref.	
No	959	86.7 (959/1106)	77.6 (737/950	0.71	0.45–1.12	.140
Assisted pregnancy such as in vitro fertilization or artificial insemination						
Yes	67	6.1 (67/1106)	85.1 (57/67)	1	Ref.	
No	1039	93.9 (1039/1106)	77.9 (802/1030)	0.62	0.31–1.23	.168
Type of health care provider as first contact in pregnancy						
Midwife	320	29.1 (320/1110)	71.3 (228/320)	1	Ref.	
General practitioner	129	11.7 (129/1110)	72.9 (94/129)	1.08	0.69–1.71	.730
Gynaecologist	609	55.4 (609/1110)	83.3 (507/609)	2.01	1.45–2.77	<.001
Others	42	3.8 (42/1110)	76.2 (32/429)	1.29	0.61–2.73	.504

**Fig. 1:**
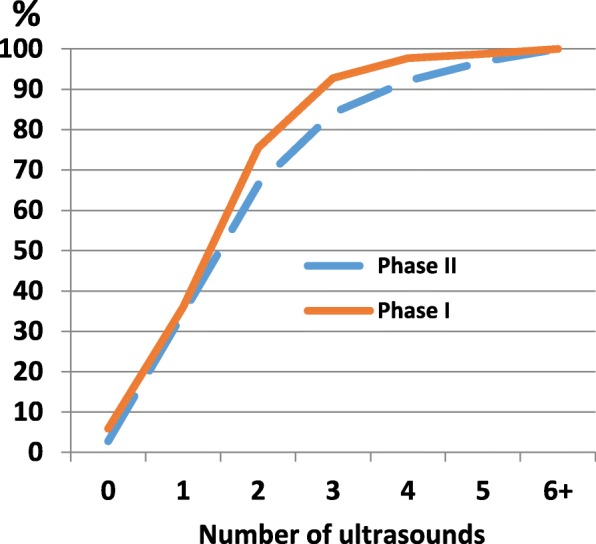
Cumulative percentage of the number of ultrasound scans before pregnancy week 19 (phase I, *N* = 1038). For validation, recalled number of ultrasounds among respondents after delivery (phase II; *N* = 763)

A total of 95% (CI 93.1–95.8%) of the pregnant women had undergone at least one ultrasound scan before the recommended scan in week 19. Figure 1 shows the cumulative percentage of the number of ultrasound scans in pregnancy. As shown, 64% underwent two or more scans, and 2% had four or more scans before the 19th week scan.

Seventy-eight percent of the women had participated in early screening for foetal anomalies in week 11–14 (Table 1). Among women above 35 years, 88% chose the screening test, as did 75% among 18–24 year-old-women. There were substantial differences in foetal screening participation rates between primipara and multipara women, women with high versus low education level, and women in urban and rural areas (Table 1).

To test potential recall bias regarding the number of early scans, we compared the number of scans reported by the same women in phases I and II. When the routine scan in week 19 was excluded from the total reported in phase II, the numbers corresponded well (Fig. 1).

After becoming pregnant, 55% of the women consulted a private gynaecologist with ultrasound equipment (Table 1). These women were subsequently more likely to choose prenatal screening in week 11–14, compared to women who attended primary care midwives or GPs as their first contact (*p* < .001).

Table 2a shows how the decision to attend or decline prenatal screening for foetal anomalies was reached. It appears that the decision was typically reached within the family, marginally influenced by a healthcare professional. Half the women reported that they found it “self-evident” to participate in this screening.

**Table 2:** Percentage (absolute figures within brackets) of the women’s main reasons for choosing or denying prenatal screening for foetal anomalies in week 11–14 of pregnancy, potential concerns related to the unborn childs` health, their experience of the screening test and the main reasons for declining the test

2a) Main reason for choosing to attend prenatal screening*	%	n/N	95% CI
My partner and I agreed on participating	74.5	636/854	0.71–0.77
To me it was self-evident to participate	49.6	424/854	0.46–0.53
Everyone I know have had the screening	4.8	41/854	0.04–0.06
Advice from doctor	4.9	42/854	0.04–0.07
The child’s father wanted me to participate	4.5	38/854	0.03–0.06
Advice from midwife	3.7	32/854	0.03–0.05
2b) Had the prenatal screening impact on your worries about whether something was wrong with the child?			
Yes, it increased my concerns	9.5	79/828	0.08–0.12
Yes, it reduced my concerns	61.5	509/828	0.58–0.65
No, the screening did not affect my concerns	18.0	149/828	0.16–0.21
I never had any concerns there was something wrong with the child	11.0	91/828	0.09–0.13
2c) How would you describe your experience with the combined test?			
Very positive	58.3	483/828	0.55–0.62
Positive	34.1	282/828	0.31–0.37
Neither positive nor negative	6.5	54/828	0.05–0.08
Negative	0.6	5/828	0.00–0.01
Very negative	0.5	4/828	0.00–0.01
2d) Why did you choose to not do the combined test? *			
Do not consider myself at being at risk of having a child with foetal anomalies	39.0	92/234	0.33–0.46
Do not think I could make a decision if there was a possibility for foetal anomalies	38.9	91/234	0.33–0.45
Do not think it is reliable enough	26.1	61/234	0.21–0.32
My values and beliefs	52.6	123/234	0.46–0.59

*More than one option possible

Table 3 shows how the participants evaluated the information they had received regarding the prenatal screening, subsequent diagnostic tests and associated risks. Most women were satisfied with the information they got about the screening programme as such, but less satisfied with the information about its potential downsides and risks. Regarding experiences of the 11–14 week screening, most women (92%) reported a positive experience (Table 2c).

**Table 3:** Perception of information given and odds (OR) for decision making regarding early prenatal NT* screening or the combined test** for foetal anomalies at week 11–14. Percentages, absolute figures within brackets, odds ratios (OR) and 95% confidence intervals (95% CI)

	Number	%	Combined prenatal screening test at week 11–14			
			%	OR	95% CI	*p*-value
Information about availability of first trimester ultrasound scan						
Enough or too much	906	84.2 (906/1076)	79.2 (718/906)	1	Ref.	
Not enough	52	4.8 (52/1076)	75.0 (39/52)	0.79	0.41–1.50	.465
No information	118	11.0 (118/1076)	72.0 (85/118)	0.67	0.44–1.04	.075
Information about availability of early prenatal screening in week 11–14						
Enough or too much	938	86.8 (938/1081)	79.6 (747/938)	1	Ref.	
Not enough	69	6.4 (69/1081)	66.7 (46/69)	0.51	0.30–0.87	.012
None	74	6.8 (74/1081)	73.0 (54/74)	0.69	0.40–1.18	.176
Information about the potential risk of early prenatal screening in week 11–14						
Enough or too much	521	48.3 (521/1078)	76.6 (399/521)	1	Ref.	
Not enough	145	13.5 (145/1078)	80.7 (117/145)	1.28	0.81–2.02	.296
No information	412	38.2 (412/1078)	80.3 (331/412)	1.25	0.91–1.72	.168
Information about the potential risk associated with foetal prenatal diagnosis						
Enough or too much	509	47.2 (509/1079)	78.0 (397/509)	1	Ref.	
Not enough	153	14.2 (153/1079)	81.7 (125/153)	1.26	0.80–2.00	.326
No information	417	38.6 (417/1079)	78.2 (326/417)	1.01	0.74–1.38	.947
Information about upcoming/routine ultrasound scan in week 19–20						
Enough or too much	815	75.5 (815/1080)	75.7 (617/815)	1	Ref.	
Not enough	152	14.1 (152/1080)	85.5 (130/152)	1.90	1.17–3.06	.009
No information	113	10.5 (113/1080)	91.2 (103/113)	3.31	1.69–6.45	<.001

*NT Foetal nuchal translucency

**ß-hcg and PAPP-A measured in plasma and NT measurements by ultrasound

As illustrated in Table 1, 22% of the included women did not attend early screening for foetal malformations. Table 2d shows the reasons women reported to underpin this decision. As shown, the main reason was their personal values and views in general, followed by not considering their pregnancy/foetus to be at-risk, reluctance to face a potential decision of whether or not to terminate the pregnancy, and doubts regarding the reliability of the test. Among women who underwent foetal screening in week 11–14, the majority (61,5%) reported reduced concerns regarding the unborn child’s health after the test, 18% reported unchanged concerns, and 9,5% reported increased concerns (Table 2b).

## Discussion

The Icelandic Childbirth and Health Study is a comprehensive primary care cohort study on pregnancy and childbirth. Although ultrasound before week 19 has so far not become part of routine antenatal care in Iceland, our data shows that 95% of all participating women had at least one scan, and 64% had two or more scans in this period. In many instances, the early scans seem to involve one consumer-initiated “confirmation scan” with a private gynaecologist, followed by the optional, early screening for foetal anomalies in week 11–14, which was chosen by 78% of the women. There were substantial differences in screening participation between subgroups. This is likely to mirror varying accessibility to secondary health care, as well as differing views of what it means to be pregnant and how to behave in this situation. The fact that primipara women attended early foetal screening more frequently than multipara women (who are likely to be older with a higher statistical risk of foetal anomalies) suggests that pregnant women’s motivations for ultrasound examinations in pregnancy are not fixed, but can vary with time, life experience and other circumstances. The complex impact foetal scanning might have on pregnant women and their partners has been discussed for decades, as can be illustrated by Lumley’s 1990 paper “Through a glass darkly” [26]. Similarly, the issue of medicalization of pregnancy has been debated for years [27]. To what extent the frequent use of ultrasound scanning found in this study represents a favourable trend, or whether it entails a tendency towards undue medicalisation of pregnancy and misuse of healthcare resources, is open for debate.

## A ‘self-evident’ test and an informed choice?

Our study indicates that the decision to attend prenatal screening in week 11–14 was mainly made within the family, and not directly initiated by a healthcare professional. The fact that many women found it “self-evident” to participate, further suggests that among substantial subgroups of women, early foetal screening has become deeply embedded in the wider culture of pregnancy. Interestingly, however, when asked about pre-screening information about potential associated risks, many found this unsatisfactory. Similar findings have been reported from Sweden [5, 28]. This might stem from lacking, suboptimal or biased pre-screening information given by the healthcare workers, but perhaps also from low receptivity to information on potential downsides of screening among the women prior to the decision to participate. Asking them later, as we did in our study, might thereby elicit some afterthoughts. Seen together, these findings lead us to question whether participation in the 11–14 week screening represented an informed, autonomous choice for the majority of the women. It would be interesting to study the information process in more detail at the different provider settings and levels. As Williams [13] has pointed out, a general criticism of prenatal screening is that the women might not realize how much technology has come to influence the culture surrounding pregnancy, nor what a genuinely free choice actually entails. The main reason our responding women gave for *declining* the early foetal screening test, was their personal values and beliefs. Secondly, many said that they did not consider themselves as “at-risk”. This is also in accordance with earlier studies on the subject [29, 30].

## Comparison with other studies

To our knowledge, this is the first study of ultrasound scanning among Icelandic women in the first half of pregnancy, until the recommended routine scan in week 19.

Data from the Icelandic Birth Registry [31] indicate that 73% of pregnant women underwent early prenatal screening in pregnancy week 11–14 in the year 2009 when this study started. This figure is slightly lower than our finding of 78%, which can partly be explained by a certain overrepresentation of highly educated women in our material [32]. It might also be that some women in our 2009 study misclassified an early clinical scan as formal screening. Recent figures from the national Birth Register in Iceland indicate that 80% of women currently attend the combined screening test [31].

We have not found international data for comparison of Icelandic women‘s use of early ultrasound in pregnancy. A Swedish study showed that 36% of pregnant women in year 2013 underwent combined screening [33]. This is much lower than in our study. A Danish study from 2008 showed that the number of women who chose early foetal screening increased from 63% in 2005 to 84% in 2006. This can probably be explained by the introduction of an offer of prenatal screening between 2004 to 2006 [34]. More recent numbers suggest that over 90% of Danish women undergo the combined test [35].

Norway currently has no established programme for early foetal screening. Experts in foetal medicine however report that a high number of pregnant Norwegian women seek out a private clinic and have an early ultrasound scan done there, not unlike the Icelandic situation [36]. If that scan is suggestive of anything abnormal, the woman is referred to an authorized department for foetal medicine. This pathway can be described as an informal ‘back door’ to early foetal screening.

## Strengths and weaknesses of the study

The main strength of our study is its size, encompassing 23% of all childbirths in Iceland in 2009. The original sample has been considered relatively representative [23, 32].

Our convenience sampling method is unlikely to have resulted in serious selection bias, as the study addressed women who attended routine antenatal care, with a focus on their general experiences, thoughts and attitudes as pregnant women in modern society. A certain response bias could, however, not be avoided, as women who ultimately answered the questionnaires are likely to have been more interested in the research topics than those who were initially positive, but did not return the questionnaire [24].

Women with higher education might be somewhat overrepresented [32]. It is a weakness that the number of ultrasound scans are self-reported, but our test of potential recall bias suggests that the overall numbers are quite reliable. Another weakness is the drop out between Phases I (before mid-pregnancy) and II (after delivery). Several factors may have contributed to this. In 2009, the financial crisis in Iceland led to substantial work-related emigration, and it is likely that some of our original participants had left the country. In addition, a few pregnancies are likely to have ended unsuccessfully, diminishing the mother’s motivation to participate in Phase II. We do not have precise data on these matters.

## Conclusions

Ultrasound scans in early pregnancy are in high use in Iceland and have apparently become a profiled part of the pregnancy culture. We found substantial variations regarding uptake of early foetal screening among subgroups. Whether the widespread use of early scanning represents a favourable development or a sign of undue medicalization and overuse of medical resources, can be debated. Information prior to prenatal screening for foetal anomalies might be improved, particularly regarding potential side effects and risks associated with the screening programme.

## Abbreviations

C&H study: Childbirth and Health Study
CI: Confidence Interval
GP: General Practitioner
NIPT: Non Invasive Prenatal Testing
NT: Nuchal Translucency
OR: Odds Ratio
PAPP-A: Pregnancy associated plasma protein A
SPSS: Statistical Package for Social Sciences
ß-hcg: ß-human chorionic gonadotropin blood test
